# Smartphone-Based Technologies in Equine Sports Medicine: Supporting Athlete Management—A Review

**DOI:** 10.3390/s26134002

**Published:** 2026-06-24

**Authors:** Federica Meistro, Paola D’Angelo, Alessandro Spadari, Riccardo Rinnovati

**Affiliations:** Department of Veterinary Medical Sciences, University of Bologna, 40064 Ozzano dell’Emilia, BO, Italy; paola.dangelo7@unibo.it (P.D.); alessandro.spadari@unibo.it (A.S.); riccardo.rinnovati2@unibo.it (R.R.)

**Keywords:** equine sports medicine, smartphone-based technologies, wearable sensors, artificial intelligence, equine welfare

## Abstract

Equine sports medicine is increasingly oriented toward objective, field-based monitoring systems that support both performance optimization and welfare assessment. In this context, smartphone-based technologies have emerged as accessible tools capable of integrating data acquisition, processing, and interpretation within a single platform. This narrative review aims to examine the role of smartphones in equine sports medicine, focusing on their function as standalone sensing devices and as gateways for wearable and external sensor systems. The analysis is based on a structured synthesis of current literature addressing technological foundations, including embedded sensors, connectivity architectures, and artificial intelligence-driven data processing, as well as their clinical applications across locomotor, cardiovascular, respiratory, behavioural, and thermoregulatory domains. Evidence indicates that smartphone-based systems improve the feasibility of longitudinal monitoring and facilitate real-time decision-making in field conditions, while enhancing communication between veterinarians, trainers, and owners. However, their performance remains influenced by acquisition conditions, system variability, and algorithmic constraints, requiring careful validation and contextual interpretation. In addition, challenges related to data governance, privacy, and ethical use remain insufficiently addressed. Overall, smartphone-based technologies represent enabling tools that support a transition toward more integrated, data-driven, and welfare-oriented management of the equine athlete, while highlighting the need for standardisation and regulatory development.

## 1. Introduction

In recent years, equine welfare has received increasing scientific and societal attention, particularly within the context of equestrian sports, where training practices, competition demands, and human expectations interact to shape the physical and mental experience of the horse [1]. Current views of welfare increasingly recognise that its assessment cannot be limited to the absence of overt disease or injury, but must also consider the animal’s physiological responses and behavioural adaptations to exercise and management conditions [2].

In the equine athlete, exercise induces complex and interdependent adaptations across multiple physiological systems, including the cardiovascular, respiratory, musculoskeletal, thermoregulatory, and endocrine systems. The coordinated function of these systems determines not only performance, but also the horse’s ability to cope with workload, recover effectively, and maintain overall physiological and behavioural balance [3,4,5]. Consequently, the quantitative monitoring of these responses has become central to both performance optimisation and welfare assessment [6].

Traditionally, equine health and performance have been assessed through clinical examination, laboratory testing, and specialised diagnostic tools. Although these approaches remain essential, they are often limited by the need for controlled environments and the difficulty of obtaining continuous or repeated measurements in field conditions [7,8].

In practical settings, the value of a monitoring system is therefore not determined solely by its technical accuracy, but by its usability and integration into daily workflows [6]. In equine sports medicine, tools must be applicable across different users—including veterinarians, trainers, physiotherapists, and owners—and must support frequent measurements, longitudinal monitoring, and real-time decision-making [9]. Early identification of subtle changes in movement, behaviour, or physiological parameters is particularly relevant, as these often precede clinically detectable conditions [10,11].

Within this evolving landscape, a progressive shift towards more accessible and field-applicable monitoring solutions has emerged, paralleling developments in human digital health. The rapid expansion of mobile health (mHealth) technologies [12] has demonstrated how portable and connected systems can enable continuous monitoring outside clinical environments, supporting more personalised and data-driven approaches to health management [13,14,15]. This paradigm is increasingly being translated into veterinary medicine, including equine sports practice [16].

In particular, this transition has been accompanied by the emergence of the smartphone as a central technological node within modern monitoring ecosystems. Rather than acting as a simple accessory, the smartphone increasingly functions as an integrative platform capable of connecting sensors, processing data, and delivering actionable information in real-time [17,18]. They can function as direct measurement tools through embedded sensors, as gateways for wearable technologies, and as platforms for video-based analysis, telemedicine, and real-time data visualisation [19,20].

Importantly, smartphone-based technologies do not necessarily introduce entirely new measurement principles, but rather enhance the accessibility and usability of existing monitoring approaches [21]. This shift is therefore not purely technological, but conceptual, enabling a reorganisation of data acquisition and interpretation workflows from isolated measurements towards integrated, continuous monitoring systems. By enabling data collection directly in the field and facilitating immediate interpretation, they support a transition from episodic assessments to more continuous and context-specific monitoring of the equine athlete [6].

Furthermore, the integration of AI and data-driven methods into smartphone-based platforms is expanding their potential applications. Automated analysis of sensor and video data, pattern recognition, and decision-support systems are increasingly incorporated into these tools, contributing to more objective and reproducible assessments [22,23].

This narrative review aims to explore the emerging role of smartphone-based technologies in equine sports medicine, with particular emphasis on their relevance in clinical and field settings. The focus is not only on the technologies themselves, but on how they reshape monitoring architectures, support day-to-day decision-making, improve longitudinal assessment of the equine athlete, and contribute to a more practical and welfare-oriented approach to performance evaluation. Publications addressing these topics were identified through a non-systematic literature search conducted in PubMed. The search strategy included combinations of the following keywords: “equine sports medicine”, “smartphone-based technologies”, “wearable sensors”, “equine monitoring”, “equine markerless motion analysis”, “artificial intelligence in equine medicine”, and “digital health in veterinary medicine”. Publications considered relevant to the scope of this review were selected based on the authors’ judgment, and their main findings were summarised using a descriptive approach.

## 2. Technological Foundations of Smartphone-Based Systems

Smartphones should not be interpreted as single-purpose sensing devices, but rather as integrated technological platforms combining multiple sensing modalities, embedded computational capabilities, and application-driven interfaces [24,25]. In equine sports medicine, their role extends beyond the use of individual sensors such as cameras or inertial units, enabling the practical deployment of mobile applications that integrate acquisition, processing, and interpretation within a single device [6]. This dual nature, hardware sensing combined with software-driven functionality, implies that the effective performance of smartphone-based systems depends not only on the characteristics of embedded sensors, but also on how these signals are accessed, processed, and interpreted within specific application contexts [17,26]. Importantly, this interaction becomes particularly relevant in equine field conditions, where measurements are influenced by environmental variability, operator-dependent acquisition, and animal-related factors [6]. Rather than replacing laboratory-grade instrumentation, smartphones should therefore be regarded as enabling tools that facilitate scalable, field-deployable, and context-rich data acquisition. Their value lies in bridging the gap between subjective clinical assessment and high-end instrumentation, supporting repeated measurements, longitudinal monitoring, and real-time decision-making in the equine athlete [6].

### 2.1. Smartphone Hardware Capabilities

#### 2.1.1. System Integration and Measurement Trade-Offs

Modern smartphones incorporate a heterogeneous set of sensing and computing components within a single, compact, battery-constrained platform. This convergence includes imaging systems, inertial sensors, microphones, positioning modules, data storage, and wireless communication interfaces, all of which contribute to their versatility in biomedical and veterinary applications [25,27]. At the same time, this convergence should not be interpreted as equivalent to purpose-built scientific instrumentation. Consumer smartphones are designed primarily for usability, energy efficiency, and mass-market performance rather than for metrological transparency, deterministic timing, or long-term signal stability [28]. As a result, their value in quantitative assessment lies less in replacing gold-standard laboratory systems than in enabling scalable, field-deployable, and context-rich measurements under real-world conditions [25,29]. This integrated nature also enables smartphones to support a wide range of application-driven monitoring strategies in equine practice. Smartphones provide an opportunity for increased accessibility and frequency of data acquisition, but require careful consideration of their limitations when used for quantitative assessment.

#### 2.1.2. Camera-Based Sensing

The camera system is the most influential hardware component for vision-based smartphone equine applications. Modern devices rely on CMOS image sensors [30] integrated with proprietary image signal processing pipelines that handle demosaicing, denoising, exposure control, stabilization, and compression [31]. These capabilities have enabled the development of vision-based applications such as digital phenotyping [32,33,34], markerless motion analysis [35,36], and pose estimation [37], all of which are directly relevant to equine sports medicine. However, from a measurement perspective, the camera is not a neutral acquisition device. Rolling shutter remains a typical characteristic of mobile imaging, meaning that the image is captured line by line rather than instantaneously across the full frame, which can induce geometric distortions when either the camera or the subject moves rapidly [38]. In particular, markerless gait analysis systems implemented via smartphone video acquisition have emerged as practical tools for field-based assessment of locomotor asymmetries [35]. From a measurement perspective, however, smartphone cameras are not neutral acquisition devices. Rolling shutter effects, where the image is captured line by line rather than instantaneously, can introduce geometric distortions during rapid motion [39]. Additional factors such as motion blur, automatic exposure adaptation, image enhancement, and compression may alter the spatiotemporal fidelity of the recorded signal [40]. These limitations become particularly relevant in equine locomotion analysis, where rapid cyclic movements, short stance phases, and subtle asymmetries require high temporal and spatial accuracy [41]. Consequently, the effective performance of camera-based smartphone applications depends not only on nominal resolution, but on the interaction between acquisition rate, optical properties, and downstream processing pipelines [42]. In practical terms, smartphone cameras may perform very well for gross movement pattern recognition and increasingly well for markerless asymmetry detection, but they remain vulnerable to systematic distortions when measurements depend on precise timing, very high-speed events, or strict geometric fidelity. This is one reason why validation against reference systems remains essential whenever a smartphone camera is used as a quantitative sensing interface rather than as a simple recording device [35,43]. While camera-based sensing is most commonly associated with locomotor assessment, its application is not restricted to a single physiological system and may extend to multiple domains of equine monitoring, as further discussed in Section 3.1.

#### 2.1.3. Inertial Sensing (IMUs)

Inertial sensing in smartphones is based on microelectromechanical systems (MEMS) integrating tri-axial accelerometers and gyroscopes, which enable the measurement of linear acceleration and angular velocity [44,45]. These sensors provide the basis for motion analysis and have been widely adopted in both human and veterinary research, particularly within wearable systems designed for field-based applications [46,47]. Despite their accessibility and versatility, smartphone-integrated IMUs present intrinsic limitations. Noise, bias instability, and thermal sensitivity can introduce drift, particularly when signals are integrated to estimate velocity, position, or orientation [48,49]. In human gait analysis, this is particularly critical, as integration errors may propagate and significantly affect higher-order kinematic parameters [50].

From an application perspective, the direct use of smartphones as inertial sensing devices in equine monitoring remains limited. Equine locomotion analysis typically requires precise sensor placement at specific anatomical landmarks (e.g., head, pelvis, limbs), stable attachment, and consistent orientation. Consequently, inertial sensing in equine sports medicine is predominantly implemented through dedicated wearable IMU systems rather than through embedded smartphone sensors [41]. However, the conceptual and technological framework remains closely related, as smartphones frequently act as data acquisition hubs, user interfaces, or processing platforms for these external sensors [50,51].

In this context, the relevance of smartphone-based inertial sensing is primarily indirect: while the embedded IMUs illustrate the principles of motion sensing, the practical deployment of inertial analysis in equine applications relies on integrated systems combining wearable sensors and smartphone-based data management [6,41]. Accordingly, smartphone IMUs should be considered enabling technologies rather than standalone measurement solutions.

#### 2.1.4. Additional Sensors (Microphone and Global Navigation Satellite System (GNSS)/ Global Positioning System (GPS))

In addition to imaging and inertial sensing, smartphones integrate further sensing modalities, including microphones and GNSS/GPS modules, which may contribute to equine monitoring under specific conditions.

The smartphone microphone adds an acoustic sensing channel that is often underappreciated. In principle, embedded microphones can support event detection, respiratory or vocal signal capture, environmental characterization, and pattern recognition [52,53]. In equine medicine, respiratory sounds have long been used to investigate upper airway function and to detect pathological conditions during exercise. Recent developments in signal processing and machine learning have demonstrated that respiratory events can be automatically detected from audio recordings in horses, enabling the estimation of breathing patterns and respiratory rate during exercise [54]. However, the practical implementation of smartphone-based acoustic sensing remains limited. Most current approaches rely on dedicated microphones positioned close to the respiratory source and are often developed under controlled or semi-controlled conditions [54]. Field-based applications remain relatively scarce, and challenges such as environmental noise, motion artefacts, and variability in sensor placement continue to affect signal quality and robustness [55]. As a result, while acoustic sensing represents a biologically meaningful and increasingly investigated approach, its integration into smartphone-based systems is still at an early stage and remains largely exploratory in equine sports medicine.

GNSS/GPS modules provide spatial and temporal information, enabling estimation of speed, distance, and movement patterns. These capabilities are particularly relevant in outdoor equine training and competition environments, where workload and movement context are important components of performance evaluation [6]. Nevertheless, the direct use of smartphone-based GNSS for equine monitoring is limited in practice. Dedicated wearable systems are typically preferred due to improved positioning stability and signal reliability. Smartphone GNSS measurements remain affected by noise, environmental obstruction, antenna limitations, and device-specific variability [56,57]. Consequently, GNSS data are best interpreted as contextual indicators of locomotor activity rather than as precise biomechanical measurements [58].

### 2.2. Connectivity Architecture

A defining characteristic of smartphone-based sensing systems is that the device rarely operates in isolation. Instead, it functions as a central coordination hub within a distributed digital ecosystem, integrating internal sensors, external devices, cloud-based services, and user interfaces [26]. This role is particularly relevant in sports medicine and veterinary monitoring, where data acquisition often depends on the combined use of inertial sensors, cameras, physiological wearables, positioning systems, and remote analytical platforms. In such contexts, the smartphone acts as the primary interface for data acquisition, synchronization, and visualization, directly influencing the usability of the system in daily practice [24].

#### 2.2.1. Short-Range Communication (Bluetooth and Ant Plus)

Bluetooth and Bluetooth Low Energy (BLE) are the most widely used communication **or** connecting wearable sensors to smartphones. Their low power consumption and broad compatibility make them suitable for continuous data acquisition in field conditions. However, BLE transmission is not inherently lossless or temporally deterministic. Packet loss, latency variability, and synchronization issues may occur depending on data rate, buffering strategies, and system configuration [59]. These factors are particularly relevant in equine applications involving multi-sensor systems, such as combined locomotor and physiological monitoring, where temporal alignment between signals is essential for meaningful interpretation (e.g., synchronizing gait asymmetry data with heart rate or workload metrics) [6].

ANT+ (Advanced and Adaptive Network Technology Plus) has historically played an important role in sports and physiological monitoring because of its lightweight communication model and interoperability across compatible sensor platforms [60]. However, BLE has become the dominant smartphone-facing protocol in most contemporary sensing workflows, largely because of broader operating system support and easier app-level deployment [59].

#### 2.2.2. Wireless Network Communication

Wi-Fi connectivity supports higher data throughput and is particularly relevant when smartphones must transmit large datasets such as high-resolution video, or support near-real-time upload for remote processing [61]. In equine sports medicine, this is especially important for markerless gait analysis systems, where video recordings acquired in the field are often processed remotely. Network availability in stables, training facilities, or competition environments may be inconsistent, limiting real-time data transmission [35]. As a result, many smartphone-based equine monitoring systems rely on hybrid strategies combining local storage with delayed synchronization, allowing data acquisition in the field and subsequent processing when connectivity is available [41].

### 2.3. Data Processing and Management

The computational role of the smartphone has evolved substantially. Earlier mobile sensing systems often treated the smartphone mainly as an acquisition and forwarding device, whereas current platforms increasingly use it as an active computational layer capable of preprocessing, inference, and interactive feedback [27,62]. As a result, smartphone-based systems now operate across three broad paradigms: on-device processing, cloud-based processing, and hybrid edge–cloud architectures.

On-device processing allows immediate output such as movement classification, event detection, or quality control feedback during acquisition [63]. This can be advantageous in clinical triage, sports screening, and field veterinary examinations, where rapid interpretation may improve usability and adoption. On-device processing also reduces dependency on stable connectivity and may improve privacy by limiting transfer of raw data to external servers [64]. For example, vision-based systems may provide rapid feedback on locomotor asymmetry directly after video acquisition, supporting real-time decision-making [65]. However, local processing remains constrained by battery consumption, thermal throttling, memory limits, and model-size restrictions.

Cloud-based processing offers the opposite profile. Offloading computation to remote servers enables more complex analytics, including large-scale data integration and advanced machine learning applications. This is particularly attractive for large-scale longitudinal monitoring and for applications requiring model retraining, cross-user benchmarking, or heavy video analysis [66,67]. In equine monitoring systems, this paradigm is commonly used for the analysis of video data, wearable sensor outputs, and longitudinal performance metrics, where computational demands exceed the capabilities of mobile devices. It also enables aggregation of data across multiple sessions, facilitating long-term tracking of locomotor, physiological, or workload-related parameters [6].

Hybrid architectures represent the most common and practical solution in equine sports medicine. In these systems, initial data acquisition and preprocessing are performed on the smartphone, while more computationally intensive analyses are carried out in remote servers. This approach is characteristic of many contemporary equine monitoring platforms, where data collected during training or clinical examination are reviewed either in near real-time or retrospectively through dedicated applications or cloud-based dashboards [6,41].

### 2.4. AI as an Emerging Component of Smartphone-Based Systems

In addition to sensing, connectivity, data processing and management, AI has emerged as a distinct and increasingly influential component of smartphone-based monitoring systems. While early mobile sensing platforms primarily relied on direct signal acquisition and deterministic processing pipelines, contemporary systems increasingly incorporate machine learning algorithms that transform raw data into higher-level representations, classifications, and clinically relevant outputs [68]. In this sense, AI should not be interpreted merely as an extension of data processing, but as an additional functional layer that fundamentally reshapes how information is generated and interpreted within smartphone-based ecosystems [69]. This transformation is also evident in equine sports medicine, where AI-driven approaches have enabled the practical implementation of various monitoring strategies [6]. Computer vision and deep learning techniques have made it possible to extract kinematic information directly from video data acquired with standard smartphone cameras, without the need for physical markers or laboratory-based motion capture systems [35,36,43,70]. These methods rely on pose estimation frameworks capable of identifying and tracking anatomical landmarks, enabling the quantification of locomotor patterns and the detection of gait asymmetries under field conditions. As a result, smartphone-based applications have evolved from simple recording tools to systems capable of providing objective locomotor assessment in real-world environments [71].

Beyond vision-based applications, AI also supports the integration and interpretation of multimodal data streams. Machine learning models can combine information derived from video, inertial sensors, and physiological monitoring systems [72] and establish treatment in common conditions, like colic [73], to generate more comprehensive representations of the equine athlete’s condition.

However, the inclusion of AI as a system component introduces additional sources of variability and uncertainty. Model performance depends on the characteristics of the training data, and may be affected by dataset bias and environmental variability typical of equine field conditions. Furthermore, many AI-based systems operate as non-transparent models, where decision pathways are not directly accessible, raising important considerations regarding interpretability and clinical trust [74].

From a measurement perspective, AI does not eliminate the inherent constraints associated with sensing technologies; therefore, validation against established reference systems and formal assessment of agreement remain essential when AI-based approaches are used for quantitative evaluation, particularly in equine locomotion analysis, where biological variability, movement complexity, and environmental conditions (e.g., surface, circular motion, handler influence) introduce additional sources of variability and potential measurement error [75,76,77,78].

Despite these limitations, the integration of AI has substantially expanded the functional scope of smartphone-based monitoring systems. By enabling automated feature extraction, scalable analysis, and near real-time feedback, AI contributes to making objective assessment more accessible and repeatable in equine practice [78].

## 3. Smartphone as a Standalone Sensor in Equine Sports Medicine

### 3.1. The Locomotor System

The locomotor system is a fundamental component in the assessment of the equine athlete, as its functional integrity and efficiency directly influence both athletic performance and overall welfare [79,80]. Lameness remains one of the most prevalent and clinically significant conditions, accounting for a substantial proportion of reduced athletic performance, interruption of training, and premature retirement in sport horses [81]. Consequently, accurate detection and quantification of locomotor asymmetry are essential components of both clinical decision-making and preventive management strategies [82].

Traditionally, lameness assessment has relied on subjective visual evaluation, in which clinicians identify asymmetries in vertical displacement patterns of the head, withers, and pelvis during locomotion [83]. Despite being deeply embedded in clinical practice and supported by standardized grading systems, this approach is inherently limited by its subjectivity [84]. Numerous studies have demonstrated considerable variability in both inter- and intra-observer agreement, particularly when assessing subtle or low-grade lameness. This variability is especially pronounced in hindlimb lameness, where compensatory mechanisms and biomechanical complexity further challenge visual interpretation [75,76,77,85,86]. These limitations have driven the development of objective gait analysis systems aimed at improving the sensitivity, repeatability, and quantification of locomotor assessment [87,88]. Optical motion capture systems are widely regarded as the gold standard, offering highly accurate three-dimensional kinematic data. However, their applicability is constrained by high costs, technical complexity, and the requirement for controlled laboratory environments, limiting their routine clinical use [78,89]. The introduction of wearable IMU systems has represented a major advance, enabling objective quantification of movement asymmetry under field conditions. These systems have been extensively validated and are now widely adopted in both clinical and research settings. Studies have demonstrated their ability to detect subtle asymmetries with high sensitivity and repeatability [90,91]. Nevertheless, IMU-based approaches are not without limitations, including the need for precise sensor placement, standardised acquisition protocols, and careful interpretation of processed data [78].

In recent years, the rapid evolution of computer vision and mobile technologies has led to the emergence of smartphone-based, markerless gait analysis systems [35]. Markerless systems extract movement data directly from video recordings by tracking anatomical landmarks over time, eliminating the need for physical markers or wearable sensors [92].

The integration of high-resolution smartphone cameras with advanced machine learning algorithms has facilitated the implementation of these systems in real-world clinical environments. Evidence supporting their validity is rapidly growing. Lawin et al. (2020) demonstrated that smartphone-based computer vision approaches can detect locomotor asymmetries with accuracy comparable to multi-camera motion capture systems [35]. Similarly, Kallerud et al. (2022) reported strong agreement between markerless and IMU-based systems, supporting the reliability of vision-based methods for assessing movement symmetry [36]. Validation studies have confirmed high levels of accuracy and precision of markerless systems when compared with three-dimensional motion capture [78].

These developments have led to the commercial availability of several smartphone applications, including Sleip^®^ (Sleip AI AB, Stockholm, Sweden) [65] and RealHorse^®^ (KeyDiagnostics ApS, Fredensborg, Denmark) [93] both of which have undergone peer-reviewed validation against IMU systems and optical motion capture technologies [35,94]. These systems utilise computer vision and AI algorithms to extract kinematic data from standard video recordings, providing a quantitative assessment of locomotor symmetry without the need for sensors or markers. Markerless gait analysis primarily focuses on the quantification of vertical displacement patterns of key anatomical landmarks, typically including the head, withers, and pelvis. From these signals, asymmetry indices are derived, such as differences between minima and maxima of vertical displacement (e.g., HDmin, HDmax for the head; PDmin, PDmax for the pelvis), which are commonly used proxies for forelimb and hindlimb loading asymmetry [35,36,95]. In clinical practice, these applications can be used in both straight-line and lungeing conditions as both conditions introduce biomechanical variations that are needed for lameness assessment.

Data acquisition is typically performed using a smartphone camera positioned at a standardised distance and orientation relative to the horse. The recorded video is processed automatically, with algorithms tracking anatomical landmarks frame-by-frame to reconstruct movement trajectories over time. Output is usually presented as quantitative asymmetry values, often expressed in millimetres, along with graphical representations of stride patterns, facilitating both immediate interpretation and longitudinal monitoring [65,95]. These systems offer significant practical advantages due to their ease of use in field conditions. Their non-invasive nature, minimal setup requirements, and rapid data processing make them particularly suited for routine clinical examinations, pre-purchase assessments, and monitoring of horses during training and competition. A key strength lies in their flexibility of use. Video recordings can be acquired directly in the field and uploaded for analysis either immediately or at a later stage, allowing asynchronous data review. This enables trainers and owners to independently record and share videos, facilitating remote veterinary assessment and supporting early detection of potential locomotor asymmetries. As a result, these systems enhance communication and collaboration between all stakeholders involved in the management of the equine athlete [95].

From a clinical perspective, their practicality makes them particularly valuable in settings where rapid and repeatable assessment is required, such as pre-race inspections [89,96] or longitudinal monitoring of performance horses [77]. Although these systems cannot replace gold-standard technologies such as optical motion capture, validation studies have demonstrated good levels of accuracy and reliability, as well as a reasonable degree of agreement with subjective clinical assessment [75,77].

Nevertheless, it is important to recognise that their outputs remain influenced by acquisition conditions and algorithmic processing, and should therefore always be interpreted within the broader clinical context [36].

In addition to Sleip^®^ and RealHorse^®^, other smartphone-based applications are commercially available, like the promising application Stride (Dr. Quentin Pleyers, Vollsjö, Sweden) [97]; however, at present, their level of independent peer-reviewed validation appears more limited compared to the aforementioned systems.

### 3.2. The Respiratory System

The assessment of respiratory function in the equine athlete remains one of the most challenging aspects of field-based monitoring, despite its critical role in performance and health. Traditional diagnostic approaches, including endoscopy and spirometry, are invasive and not suitable for routine field monitoring [98]. However, the respiratory system is still underrepresented in field monitoring technologies [6].

Recent developments have explored the possibility of extracting respiratory dynamics from video recordings acquired using standard smartphone cameras. These approaches are based on the analysis of subtle motion features associated with breathing, such as nostril dilation and thoracoabdominal excursions, which can be tracked over time to reconstruct respiratory cycles. In this context, systems such as EquiBreathe app have been developed to apply AI-based analysis of smartphone video recordings that can support the identification of horses affected by equine asthma, showing promising classification performance in distinguishing affected and non-affected individuals under controlled conditions [99]. These findings suggest that vision-based approaches may provide a feasible, non-invasive screening tool for respiratory dysfunction in the field.

Compared to alternative technologies, such as acoustic monitoring systems that rely on microphones positioned close to the nostrils [54], video-based methods offer practical advantages. However, the accuracy of video-based respiratory assessment is influenced by recording conditions, including camera positioning, movement artefacts, and lighting. In addition, respiratory motion signals may be subtle and are affected by multiple physiological and environmental factors, such as exercise intensity, body condition, and ambient conditions, which can complicate interpretation. Furthermore, the current body of evidence remains limited, with most studies conducted on relatively small populations and under controlled experimental conditions [99]. As highlighted in recent literature [6], the respiratory system is still underrepresented in equine monitoring technologies compared to locomotor assessment, reflecting both technical challenges in signal extraction and the complexity of defining robust clinical thresholds for respiratory dysfunction.

### 3.3. Behavioural System and Pain Assessment

The assessment of behavioural responses and affective state in the equine athlete has become increasingly relevant in both clinical and performance contexts, reflecting the growing recognition that welfare and performance are closely interconnected [2]. In contrast to purely physiological systems, behavioural assessment aims to capture the horse’s subjective experience, including pain, discomfort, stress, and emotional responses to training and competition [100]. Traditionally, behavioural evaluation relies on structured observational frameworks such as ethograms, which describe specific postures, movements, and behavioural patterns [7,101]. Among these, facial expression-based scoring systems have gained particular attention. Tools such as the Horse Grimace Scale (HGS) [102] and related frameworks, like the Equine Pain Face evaluation [103] and the EquiFACS [104], have demonstrated that specific facial feature, including orbital tightening, ear position, and tension in the muzzle, are associated with pain states in horses. However, despite their biological validity, these systems remain fundamentally subjective, as they rely on human interpretation.

In response to these limitations, recent developments in computer vision and machine learning have enabled the transition toward more objective behavioural assessment. Several studies have demonstrated that facial expression analysis can be automated using image-based approaches. Lencioni et al. developed a deep learning model capable of recognising pain-related facial features in horses from images, demonstrating that automated systems can achieve meaningful classification performance [105]. Similarly, Andersen et al. explored the use of machine learning for the recognition of facial expressions associated with pain, highlighting the feasibility of automated detection in horses [106]. More recent work has further expanded this field, demonstrating that computer vision models can identify emotional states from equine facial images, reinforcing the potential of image-based behavioural phenotyping [107].

These approaches are particularly relevant in the context of smartphone-based sensing, as they rely on image acquisition that can be directly performed using the smartphone camera. Behavioural signals can be captured non-invasively through video or photographs, making them inherently compatible with standalone smartphone use. This compatibility has led to the development of several smartphone applications designed to support behavioural and pain assessment in practice. For example, the Horse Grimace Scale App [108], developed within the Animal Welfare Indicators (AWIN) project, provides structured training and standardized scoring of equine facial expressions, based on the validated HGS framework. In parallel, the Equine Pain and Welfare App [109] expands this approach by integrating facial expression scoring with a broader set of welfare indicators, enabling a more comprehensive assessment of the animal’s condition and allowing longitudinal tracking of behavioural parameters over time. These tools represent an important step toward standardisation, enabling users to apply validated scoring systems consistently and to document behavioural observations over time. However, it is important to note that most currently available applications rely on user-guided scoring, rather than fully automated analysis of passive video input. Fully automated systems, based on real-time computer vision, remain largely in the research domain.

### 3.4. The Ocular System

The assessment of ocular health in the equine athlete is clinically relevant, as ocular disorders can significantly affect comfort, behaviour, and visual function. Conditions such as uveitis, corneal lesions, and conjunctival inflammation are relatively common and may influence not only the horse’s well-being but also its responsiveness during training and competition [110,111]. Traditional ophthalmological evaluation relies on specialised equipment that is not always readily available in field conditions, particularly in ambulatory practice or training environments. This creates a need for accessible, non-invasive screening tools. In this context, smartphone-based high-resolution cameras allow the acquisition of detailed images of the eye and periocular region. These images can be used for documentation, remote consultation, and, increasingly, automated analysis. In line with these developments, smartphone technology can be effectively implemented in equine clinical ophthalmology under field conditions. Specifically, the use of smartphone cameras—when combined with appropriate illumination and positioning—enables the acquisition of diagnostically useful images of the anterior segment, facilitating clinical documentation, case monitoring, and teleconsultation [112].

Recent advances in computer vision and machine learning have enabled the development of systems capable of analysing ocular images for diagnostic purposes. A study investigating automated classification of equine ocular images demonstrated that machine learning models applied to smartphone-acquired images could distinguish between normal and pathological conditions, including uveitis [113]. This represents a significant step toward objective ocular assessment, as it moves beyond simple visual documentation to the extraction of clinically relevant information directly from the image.

### 3.5. Other Clinical Applications

In addition to the aforementioned system, smartphone-based imaging is also widely used in equine practice for clinical documentation, particularly in dermatological and wound management contexts.

Photographic recording of skin lesions, wounds, and surgical sites is routinely performed in field conditions and represents an important tool for communication between veterinarians and horse owners. Studies in equine wound management have demonstrated that photographic assessment can be used to monitor healing progression, although standard two-dimensional imaging provides limited quantitative information compared to more advanced imaging approaches. For example, photogrammetric techniques have been shown to improve the precision of wound measurements compared to standard photographic evaluation, highlighting both the potential and current limitations of image-based monitoring [114].

Similarly, imaging of the hoof and distal limb is frequently performed using smartphone cameras to document conformation, hoof wall defects, and farriery interventions. However, there is a lack of peer-reviewed evidence supporting the efficacy of many farriery interventions, compounded by the fact that farrier journals are not currently peer-reviewed and that access to peer-reviewed equine literature among farriers remains limited [115]. Applications such as HoofmApp (Mustad Hoofcare Group, The Hoofcare People, Lelystad, Netherlands) [116] and the newly released Orion Horse Shoes (Orion Horse Shoes SA, Crassier, Switzerland) [117] provide structured frameworks for assessing hoof proportions and recording morphological features. However, these tools are not scientifically validated and generally rely on manual input or visual interpretation rather than fully automated measurement.

**Figure 1:**
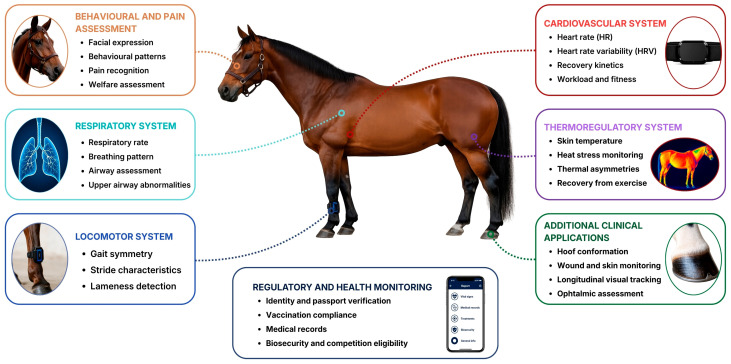
**Physiological systems and parameters monitorable in the equine athlete using smartphone-based technologies**. The figure provides an overview of the main physiological and clinical domains that can be assessed using smartphone-based technologies in equine sports medicine. These include the locomotor, cardiovascular, respiratory, thermoregulatory, and behavioural systems, as well as additional clinical applications and regulatory monitoring. Representative parameters for each domain are illustrated, such as gait symmetry, heart rate and variability, respiratory patterns, and skin temperature.

**Figure 2:**
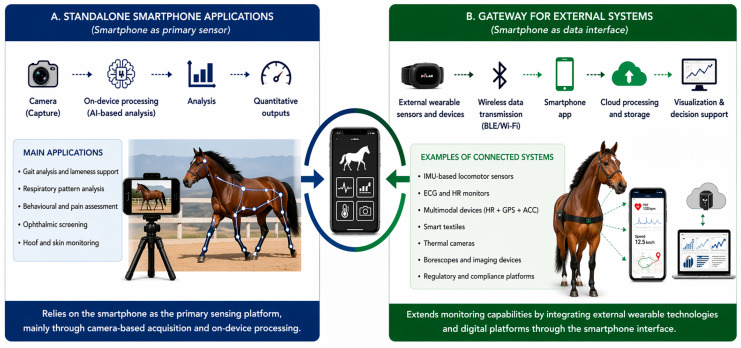
**Two complementary paradigms of smartphone-based monitoring in equine sports medicine**: Smartphone-based monitoring can be implemented either as a standalone solution (**A**), where the smartphone itself acts as the primary sensing platform, or as a gateway for an external system (**B**), enabling the integration of multiple wearable devices and digital platforms for comprehensive data acquisition analysis, visualization, and decision support.

A key distinction must therefore be made between image-based documentation and true sensing systems. While smartphone imaging is widely adopted across multiple clinical domains, only a subset of applications currently enables the extraction of objective or semi-automated information directly from the acquired signal. Nevertheless, the widespread use of smartphone imaging in these contexts highlights its practical value in field-based equine practice. Its accessibility, ease of use, and ability to support longitudinal monitoring make it an important component of modern clinical workflows, even in the absence of advanced analytical capabilities. All the systems considered in this section are summarised in Table 1, Figures 1 and 2.

## 4. Smartphone as a Gateway for Wearable and External Sensor Systems in Equine Sports Medicine

### 4.1. The Locomotor System

Building on the considerations presented in Section 3, particularly the limitations of subjective lameness assessment and the low inter- and intra-observer agreement, the development of objective locomotor analysis systems has become a critical advancement in equine sports medicine. IMU-based systems are currently considered the reference methodology for objective gait assessment under field conditions [41]. Among these, optical motion capture systems (e.g., Qualisys^®^ (Qualisys AB, Gothenburg, Sweden) [118]) and systems like the Lameness Locator^®^ (Equinosis LLC, Columbia, MO, USA) [41] remain the gold standard for high-precision kinematic analysis. However, their use is largely restricted to controlled environments due to cost, technical complexity, and limited portability.

These limitations have driven the development of more accessible, cost-effective, and field-applicable systems that can support objective locomotor assessment in routine clinical settings. Numerous commercial implementations are currently available, all based on similar sensing principles but differing in sensor configuration, data processing, and user interface design. For example, EquiGait^®^ (EquiGait Ltd., Bristol, UK) is designed to quantify vertical head and pelvic asymmetry through a dual-sensor configuration, providing stride-by-stride analysis of displacement-derived asymmetry parameters [119]. Similarly, Equi-Pro^®^ (Inertia Technology B.V., Enschede, The Netherlands) integrates IMU data into a mobile application environment, offering real-time visualization of gait symmetry and temporal stride characteristics, with an emphasis on ease of use in clinical field conditions [120,121]. Move Pro^®^ (Alogo Swiss Technology, Renens, Switzerland) adopts a comparable architecture but extends functionality toward performance monitoring, incorporating additional motion-related metrics and longitudinal data tracking features within its smartphone interface [122]. Recent developments have extended this paradigm through the introduction of limb-mounted sensors and instrumented wearable structures, such as smart boots (e.g., Tendiboots™ (Equine Therapy Solutions Ltd., Milano, Italia) and other emerging platforms), designed to capture distal limb kinematics and, in some cases, load-related parameters [123].

Despite these advances, several methodological and clinical challenges remain. First, multi-sensor systems are inherently sensitive to differences in sampling frequency, signal drift, and potential desynchronization, all of which may affect data integrity. Second, substantial variability exists in proprietary signal processing pipelines, asymmetry metrics, and threshold definitions across systems, limiting direct comparability even when similar hardware configurations are used [78,123]. This lack of standardization has been highlighted in comparative studies showing only partial agreement between different objective gait analysis platforms. These technologies should be considered decision-support tools rather than diagnostic replacements, with their outputs interpreted in relation to examination conditions, gait modality (straight-line vs. lungeing), and individual horse variability inertial [41].

### 4.2. The Cardiovascular System

The cardiovascular system represents a second fundamental pillar of equine athletic performance. Consequently, accurate and repeatable assessment of cardiovascular function is essential not only for performance evaluation, but also for training optimization and early detection of fatigue or subclinical dysfunction. The development of wearable, sensor-based systems capable of providing continuous and reproducible cardiovascular monitoring under field conditions has become a key objective in equine sports medicine [6]. Cardiovascular monitoring focuses primarily on physiological parameters such as heart rate (HR), heart rate variability (HRV), and, more recently, derived indicators of workload and recovery. These parameters are widely used to assess fitness, training response, and early signs of fatigue or overtraining in equine athletes [124]. Wearable cardiovascular monitoring systems typically rely on electrocardiographic (ECG) or photoplethysmographic (PPG) technologies integrated into girth-based or electrode-based devices [125]. Commercial systems such as Polar Equine^®^ (Polar Electro, Oy, Kempele, Finland) [126], Equimetre^®^ (Arioneo, Paris, France) [127], and other telemetric heart rate monitors are designed to continuously record HR during exercise, transmitting data wirelessly to smartphone applications for real-time visualization and storage. In particular, Polar Equine^®^ systems use electrode-based sensors positioned under the girth to capture ECG-derived heart rate, providing reliable measurements during dynamic exercise conditions [126]. Equimetre^®^ combines ECG-derived heart rate data with accelerometry and GPS measurements within a single wearable device. Through smartphone connectivity, data are transmitted in real time and processed via dedicated mobile applications and cloud-based platforms. This multimodal integration enables simultaneous assessment of locomotor activity (e.g., speed, stride-related metrics), cardiovascular response (heart rate and recovery dynamics), and workload indicators, providing a comprehensive overview of exercise physiology. Such systems are increasingly used in performance monitoring, as they allow correlation between mechanical output and physiological response, supporting more informed training decisions [127].

More advanced solutions include smart textile-based systems such as Skiin Equine^®^ (Myant Inc., Toronto, ON, Canada), which integrate conductive fibres and embedded electrodes directly into wearable fabrics. These systems enable continuous ECG signal acquisition without the need for traditional adhesive electrodes, improving ease of use and reducing preparation time under field conditions. By maintaining stable skin contact over extended periods, smart textiles facilitate the recording of inter-beat intervals (IBIs), allowing the derivation of HRV metrics and providing insight into autonomic nervous system regulation. When paired with smartphone-based applications, these systems allow real-time visualization of heart rate trends as well as storage of high-resolution temporal data for subsequent analysis [128]. Smartex^®^ (Smartex srl, Pisa, Italy) is a smart textile band with embedded electrodes and an integrated transmitter with an accelerometer that records a single-lead ECG signal for QRS complex detection and inter-beat interval analysis at a sampling frequency of 250 Hz, interfacing with applications on smartphones, computers, or tablets and primarily used under resting conditions [129], whereas Hylofit^®^ (Hylonome LLC, Wilton, CT, USA) consists of a wearable band and transmitter designed to monitor heart rate and inter-beat intervals via a smartphone application, with unspecified technical parameters, and can be used both at rest and during exercise [130].

Portable ECG devices such as AliveCor^®^ (Veterinary Heart Monitor; AliveCor, San Francisco, CA, USA) [131] and eKuore^®^ (Chip Ideas Electronics SL, Spain) [132] further illustrate the expanding role of smartphones in mobile cardiac diagnostics. These systems typically consist of compact, single-lead ECG sensors that interface directly with smartphone applications, allowing rapid acquisition and visualization of cardiac electrical activity. While originally developed for human medicine, they have been explored in equine applications for the detection of arrhythmias and basic rhythm assessment. However, their use during exercise remains limited due to motion artefacts and challenges in maintaining stable electrode contact, making them more suitable for resting or post-exercise evaluation rather than continuous monitoring during high-intensity activity. Recent developments have also introduced smartphone-based digital stethoscope systems (Eko DUO ECG  +  Digital Stethoscope, Eko Devices Inc., Oakland, CA, USA) that combine phonocardiography with simultaneous ECG acquisition. These devices allow concurrent recording of cardiac sounds and electrical activity, providing a more comprehensive assessment of cardiac function. In a recent study, such systems demonstrated almost perfect agreement with conventional auscultation and reference ECG in the detection of murmurs and arrhythmias, highlighting their potential as practical, field-applicable tools for integrated cardiac evaluation [133].

### 4.3. The Respiratory System

Despite the critical role of the respiratory system in equine performance and health, the availability of smartphone-integrated technologies for respiratory monitoring remains notably limited. As highlighted in the literature, the respiratory system is still one of the least represented domains in field-applicable monitoring tools, largely due to the inherent challenges associated with measuring respiratory function in a moving horse [6]. Unlike cardiovascular or locomotor parameters, respiratory signals are more difficult to capture reliably under field conditions. Movement artefacts, environmental noise, and the anatomical characteristics of the horse complicate both signal acquisition and interpretation. As a result, few wearable or smartphone-integrated systems have reached a level of maturity comparable to those available for other physiological systems [1].

However, recent advances in portable diagnostic imaging have introduced novel applications in which the smartphone functions as an interface for respiratory evaluation.

One of the most relevant examples is the use of smartphone-connected flexible borescopes for the assessment of the upper respiratory tract. A recent study demonstrated that a low-cost, steerable borescope connected directly to a smartphone can be used to visualise key anatomical structures of the upper airway, including the pharynx, larynx, and proximal trachea. In this study, the smartphone-based system was compared with a standard veterinary endoscope and showed good agreement in the grading of several clinically relevant conditions, including pharyngeal lymphoid hyperplasia and recurrent laryngeal neuropathy. These findings suggest that smartphone-integrated imaging systems may provide a practical alternative for field-based respiratory evaluation, particularly in settings where conventional endoscopy is not available. Nevertheless, important limitations must be considered. The absence of a working channel restricts the ability to perform sampling or therapeutic procedures, and image quality may be inferior to that obtained with standard endoscopes. In addition, mechanical limitations of the device, such as reduced manoeuvrability and durability, may affect reliability [134].

### 4.4. The Thermoregulatory System

Monitoring thermoregulation in the equine athlete may help in understanding the physiological responses to exercise, particularly under conditions of heat stress, where excessive thermal load may impair performance and compromise welfare [135]. It may also serve as a valuable tool for detecting areas of increased warmth in the horse, potentially aiding in the early identification of pathological changes [136]. In this context, smartphone-integrated systems are primarily based on the use of external infrared imaging devices, which connect directly to mobile platforms and enable real-time visualization of thermal data. These systems typically consist of portable thermal cameras—such as handheld IR sensors—coupled with dedicated smartphone applications that allow image acquisition, processing, and storage. The smartphone thus acts as both an acquisition interface and a data management platform, facilitating field-based assessment without the need for specialised infrastructure [136,137]. Infrared thermography has been widely investigated in horses as a non-invasive method to assess skin temperature and detect changes associated with exercise, inflammation, or vascular responses. Smartphone-based thermal imaging systems extend this approach by improving accessibility and enabling rapid deployment in field conditions. The ability to capture and store thermal images longitudinally also supports monitoring of changes over time and communication between clinicians and trainers. However, the interpretation of thermographic data in horses is subject to significant limitations. Skin temperature is strongly influenced by environmental factors, including ambient temperature, wind, solar radiation, and humidity. As a result, several studies have reported poor agreement between skin temperature measured by infrared thermography and core body temperature, limiting its use as a proxy for internal thermal status [138]. Similarly, a comparison between non-contact infrared thermometers with rectal temperature measurements demonstrated low reliability, further highlighting the challenges associated with surface temperature assessment in horses [139].

### 4.5. The Smartphone as a Regulatory and Monitoring Hub

In recent years, the smartphone has transitioned from a simple communication tool to a central component of digital infrastructures in equine sports medicine. Its role now extends well beyond data visualization, encompassing regulatory compliance, health monitoring, and real-time clinical decision-making. This transformation is particularly evident within the framework established by the Fédération Équestre Internationale (FEI) (Fédération Équestre Internationale, Lausanne, Switzerland), where mobile technologies have been progressively integrated into veterinary governance and biosecurity workflows [140]. One of the most significant developments concerns the digitization of equine identification systems. Historically based on paper passports, horse identification is increasingly managed through digital platforms accessible via smartphones. The FEI HorseApp represents a key example of this transition, allowing real-time access to horse identity, ownership, and competition eligibility data. Through such systems, veterinary officials can verify microchip-linked identity, review medical and vaccination records, and ensure regulatory compliance directly at the point of care. This shift enhances traceability and reduces administrative errors, while also addressing long-standing concerns related to document integrity and fraud [141].

The integration of smartphones has also had a substantial impact on vaccination monitoring and biosecurity management. Infectious disease control in equine populations, particularly for equine influenza, depends on strict adherence to vaccination schedules [142]. Mobile-based systems now enable automated validation of vaccination intervals, real-time identification of non-compliance, and standardized interpretation of regulatory requirements across different jurisdictions. Within FEI competitions, this functionality is critical for ensuring uniform enforcement of vaccination rules and minimizing the risk of disease transmission during international horse movements [143]. Moreover, the centralization of digital records facilitates longitudinal health tracking and supports the development of surveillance systems aligned with global animal health strategies [144].

All the systems considered in this section are summarised in Table 2, Figure 1 and Figure 2.

### 4.6. Measurement Accuracy Across Physiological Domains

Although smartphone-based technologies have demonstrated considerable potential across multiple areas of equine sports medicine, their measurement accuracy varies substantially according to the physiological parameter being assessed and the sensing modality employed (Table 3).

Currently, the strongest validation evidence is available for locomotor and cardiovascular monitoring applications. Markerless gait-analysis systems and wearable inertial sensor platforms have shown good agreement with established objective assessment methods, including optical motion capture and sensor-based gait analysis systems [35,36,43,75,76,77,78]. Similarly, ECG-based wearable devices and smart textile systems have demonstrated good agreement with conventional electrocardiographic recordings for heart rate monitoring and, to a lesser extent, heart rate variability assessment [126,127,128,129,130,131,132,133].

In contrast, respiratory, thermographic, behavioural, and ophthalmic applications remain at earlier stages of development. Respiratory monitoring is particularly challenging because signal quality may be affected by environmental noise, motion artefacts, and sensor positioning [54,99]. Thermographic systems provide useful information regarding surface temperature distribution and thermal asymmetries, but several studies have reported limited agreement between skin temperature and core body temperature measurements, highlighting the influence of environmental conditions on data interpretation [137,138,139]. Similarly, behavioural and facial-expression-based applications have shown promising results, although their performance remains dependent on image quality, dataset characteristics, and algorithm training [105,106,107].

These differences indicate that smartphone-based monitoring technologies should not currently be considered equally mature across all physiological domains. While locomotor and cardiovascular applications already provide clinically useful and relatively well-validated information, further validation and standardisation are still required for several emerging applications before widespread clinical implementation can be recommended.

## 5. Ethical, Data and Practical Considerations

The rapid adoption of smartphone-based technologies in equine sports medicine introduces a complex set of challenges that extend well beyond technical performance and measurement accuracy. As these systems evolve from episodic assessment tools to continuous, data-driven monitoring platforms, they reshape not only how data are generated and interpreted, but also how responsibility, ownership, and ethical accountability are conceptualised within the equestrian domain [145]. Unlike traditional diagnostic approaches, smartphone-based systems operate within distributed digital ecosystems, where data are continuously collected, remotely processed, and algorithmically transformed into clinically relevant outputs. This shift introduces a multilayered structure of data generation and use, in which technological infrastructures, software developers, and platform providers become integral actors alongside veterinarians and equestrian professionals. As a consequence, the integration of these technologies raises interconnected issues related to data governance, privacy, algorithmic transparency, and ethical responsibility [146], all of which remain only partially addressed in current equine practice and research.

Beyond issues of technical performance and validation, the practical implementation of smartphone-based technologies remains an important consideration. Although smartphones themselves are widely available and may reduce barriers to digital monitoring, the overall accessibility of these systems varies substantially according to the specific technology involved [147]. Markerless video-based applications generally require minimal additional equipment and may therefore represent one of the most accessible solutions for field veterinarians. In contrast, wearable sensor systems, smart textiles, thermal imaging devices, and cloud-based monitoring platforms may involve additional acquisition costs, subscription fees, specialised hardware, or infrastructure requirements. Furthermore, the effective use of these technologies often depends on reliable internet connectivity, data storage capabilities, and user familiarity with digital workflows [70,89,94]. These factors may represent practical limitations in ambulatory practice or remote field environments, where infrastructure and technical support are not always readily available. Consequently, successful implementation depends not only on technological performance but also on economic sustainability, ease of use, and integration into routine veterinary practice.

An additional consideration concerns the risk of overinterpretation of data generated by smartphone-based monitoring systems and AI-driven analytical tools. The increasing availability of objective measurements may create the perception that numerical outputs are inherently more reliable than clinical judgement [148,149]. However, many smartphone-based systems remain influenced by acquisition conditions, biological variability, algorithmic assumptions, and limitations in training datasets. Consequently, quantitative outputs should not be interpreted as definitive diagnoses or used in isolation to guide management decisions. This issue is particularly relevant in equine sports medicine, where monitoring results may influence decisions regarding training intensity, competition fitness, return-to-sport strategies, and welfare assessments. Excessive reliance on automated outputs may lead to inappropriate management decisions if the broader clinical context is not adequately considered [150]. Therefore, smartphone-based technologies and AI algorithms should be regarded as decision-support tools that complement, rather than replace, veterinary expertise, clinical examination, and professional judgement. Maintaining this balance is essential to ensure that technological innovation contributes positively to both equine welfare and evidence-based clinical decision-making.

### 5.1. Data Ownership and Governance

Among the most critical—and currently underdeveloped—dimensions of digital monitoring in equine sports medicine are the question of data ownership and governance. Smartphone-based systems are typically built upon cloud-based infrastructures, where data are collected through mobile devices, transmitted via wireless networks, and stored and processed across distributed servers [66]. Within this architecture, data are no longer confined to a single point of control. Instead, they circulate across a network of actors, including veterinarians, trainers, riders, owners, technology providers, and, increasingly, regulatory organisations. This results in a multi-stakeholder data ecosystem, where the boundaries between data producer, data controller, and data end-user become blurred [151]. In human healthcare, such complexity is addressed through structured regulatory frameworks, most notably the General Data Protection Regulation (GDPR), which defines roles, responsibilities, and rights related to data processing and ownership. In contrast, the equestrian sector lacks an equivalent, harmonised framework. As a result, data governance practices are often implicit, platform-dependent, and inconsistently regulated [152]. From a governance standpoint, several critical questions remain unresolved: Who retains ultimate ownership of data generated during monitoring sessions? To what extent do platform providers acquire rights over stored and processed data? Under which conditions can data be shared, transferred, or monetised? How should consent be conceptualised in a multi-actor, animal-centred context?

### 5.2. Data Privacy and Security

Closely linked to ownership and governance is the issue of data privacy and cybersecurity, which represents a critical vulnerability in smartphone-based monitoring systems. These technologies rely heavily on wireless communication protocols (e.g., Bluetooth, Wi-Fi) and cloud-based storage infrastructures, both of which are inherently exposed to security risks [153]. Although equine data do not fall under the same legal protections as human health data, they may nonetheless contain highly sensitive and commercially relevant information, particularly in professional and competitive contexts. Detailed records of performance, training intensity, locomotor asymmetries, and recovery patterns may provide insights into a horse’s condition that are strategically valuable and potentially exploitable by competitors [1,154]. Evidence from human digital health and IoT systems highlights the breadth and severity of these risks. Cybersecurity analyses have identified vulnerabilities related to data interception, unauthorised access, and manipulation of connected health systems [155]. Given the technological overlap between human and equine monitoring systems, these vulnerabilities are directly applicable to the equestrian context. However, the relative absence of regulatory oversight in equine applications may exacerbate the risk, as security standards are often determined by individual developers rather than enforced through sector-wide regulations. Ensuring data security, therefore, requires a multi-layered approach, including robust encryption during data transmission and storage, secure authentication and user access control, transparent data handling and storage policies, and clear accountability in the event of data breaches.

Failure to adequately address these aspects may not only compromise data integrity but also erode stakeholder trust, ultimately hindering the adoption and long-term viability of smartphone-based monitoring technologies [156].

### 5.3. Ethical Implications for Equine Welfare

The ethical dimension of smartphone-based monitoring in equine sports medicine is complex and inherently ambivalent. On one hand, these technologies offer significant potential to enhance equine welfare, particularly through the early detection of subclinical abnormalities, objective monitoring of workload, and more informed management decisions [6]. By enabling continuous and quantitative assessment, smartphone-based systems align with the principles of preventive and evidence-based veterinary medicine, potentially reducing injury risk and improving long-term health outcomes in equine athletes [1,89].

Broader reflections concern the “social licence to operate” in equestrian sports, where public acceptance of animal use in competition is increasingly contingent on demonstrable welfare standards and transparency in management practices. As highlighted by Heleski et al. (2020), societal expectations are shifting toward greater accountability in the use of animals in sport, requiring clear evidence that welfare is prioritised over performance outcomes [153]. Similarly, Campbell (2021) emphasises that ethical frameworks in equestrian sport must balance performance objectives with the intrinsic value of the horse, recognising the animal not merely as an athlete but as a sentient being with independent welfare needs [154]. Within this context, technology should not be viewed as value-neutral. Instead, its implementation must be guided by a welfare-centred ethical framework, in which data are used to support responsible and context-aware decision-making rather than to justify increased performance demands. This includes careful interpretation of monitoring outputs, consideration of individual variability, and integration of objective data with clinical expertise and behavioural assessment [1].

Ultimately, the ethical impact of smartphone-based monitoring systems will depend not only on their technical capabilities but also on how they are embedded within the cultural, regulatory, and professional practices of equestrian sport. When used within a welfare-oriented framework, these technologies have the potential to support more transparent, evidence-based, and ethically responsible management of the equine athlete. Conversely, without appropriate safeguards, they risk reinforcing performance-driven paradigms that may conflict with long-term welfare objectives [7].

## 6. Future Perspectives

The rapid evolution of smartphone-based technologies in human digital health provides a valuable framework for anticipating future developments in equine sports medicine. Current innovation in smartphone-based health technologies is occurring predominantly within the human medical sector. Therefore, the examples discussed below are drawn largely from the human digital health literature and are presented as potential translational developments that may shape future applications in equine sports medicine.

One of the most promising areas is the integration of biochemical sensing with smartphone platforms. In human sports medicine, portable lactate analysers connected to mobile devices are increasingly used to assess metabolic responses during exercise, providing real-time insights into anaerobic threshold and training adaptation [155]. More recently, advances in microfluidics and biosensor technology have enabled the development of smartphone-compatible systems capable of analysing small volumes of biological fluids, including sweat, saliva, and capillary blood. These systems often rely on optical detection or electrochemical sensing coupled with smartphone-based image processing or signal acquisition [156]. Although such technologies are not yet widely implemented in equine practice, their translation appears feasible. The ability to perform field-based lactate measurement through smartphone-integrated systems could significantly enhance training monitoring in equine athletes, allowing real-time assessment of metabolic workload without the need for laboratory infrastructure. Similarly, the development of minimally invasive or non-invasive biosensors may enable continuous monitoring of biochemical markers associated with fatigue, stress, and recovery, expanding the scope of physiological assessment beyond current cardiovascular metrics.

Another emerging direction is the use of smartphone-based microscopy and imaging systems. In human and veterinary diagnostics, smartphone-adapted microscopes have been developed for point-of-care analysis, enabling rapid evaluation of blood smears, cytology samples, and parasitological specimens [157]. These systems typically combine low-cost optical attachments with smartphone cameras and AI-based image analysis, allowing automated classification and quantification. In equine sports medicine, such approaches could facilitate field-based diagnostic screening, including haematological assessment, detection of inflammatory processes, or monitoring of infection status, particularly in settings where access to laboratory facilities is limited [1,6,7].

Despite these advances, several physiological systems remain underrepresented in current equine monitoring technologies, particularly the respiratory and endocrine systems. As discussed previously, respiratory assessment in the field remains technically challenging, and current smartphone-based solutions are largely limited to exploratory video or acoustic analysis [6]. Future developments may involve the integration of wearable respiratory sensors or advanced signal processing approaches capable of extracting robust respiratory metrics under dynamic conditions [158]. Similarly, endocrine monitoring, such as the assessment of stress-related hormones (e.g., cortisol), remains largely confined to laboratory analysis. The development of portable, smartphone-integrated biosensors could enable more accessible evaluation of stress and welfare-related parameters in real time [6].

## 7. Conclusions

Smartphone-based technologies are reshaping equine sports medicine by enabling accessible, field-based monitoring of the equine athlete. Their main value lies in supporting scalable and context-aware data acquisition, bridging the gap between subjective clinical assessment and objective measurement. As both standalone tools and gateways for wearable systems, smartphones facilitate longitudinal monitoring and real-time decision-making across multiple physiological domains.

The integration of AI further enhances their analytical capabilities; however, measurement variability, environmental influences, and limited standardisation require cautious interpretation. These systems should therefore be considered decision-support tools rather than diagnostic replacements.

In addition, challenges related to data governance, privacy, and ethical use remain significant. When applied within a welfare-oriented framework, smartphone-based technologies have the potential to improve early detection, clinical decision-making, and overall management of the equine athlete. Future developments are still required and should expand to cover additional aspects of equine health, with particular emphasis on validation, standardisation, and responsible integration into clinical practice.

## Figures and Tables

**Table 1:** Smartphone-based standalone sensing in equine monitoring. Only applications in which the smartphone functions as the primary sensing device are included, predominantly through camera-based acquisition. The table highlights the current distribution of technologies across physiological and behavioural domains, as well as their level of development. Maturity levels reflect a qualitative assessment based on the availability of peer-reviewed validation studies, degree of clinical or field adoption, and technological development/commercial availability. Technologies classified as High are supported by multiple validation studies and are routinely used in clinical or sporting contexts. Moderate technologies have undergone preliminary validation and show increasing practical adoption, but require additional evidence or wider implementation. Emerging technologies are characterised by limited validation data, early-stage implementation, or restricted use in research settings. Early technologies remain largely exploratory and require substantial further validation before routine clinical application.

Domain	Standalone Smartphone Modality	Main Application	Examples (Available Systems)	Maturity Level
Locomotor system	Camera-based video analysis	Markerless gait analysis, movement asymmetry detection, lameness support	Sleip AI^®^; Stride; RealHorse^®^ [35,36,65,93,94,95,96,97]	High
Respiratory system	Camera-based video analysis	Breathing pattern analysis, respiratory abnormality screening	EquiBreathe^®^; smartphone-based respiratory AI tools [99]	Emerging
Behavioural and pain assessment	Camera-based image/video analysis	Facial expression analysis, behavioural and pain assessment	Horse Grimace Scale App; EPWA [102,103,104,105,106,107,108,109]	Moderate
Ophthalmic assessment	Camera-based clinical imaging	Screening and classification of visible ophthalmic abnormalities	Smartphone-based ophthalmic AI tools [112,113]	Early
Additional visual clinical applications	Camera-based imaging	Hoof conformation documentation, wound/skin monitoring, longitudinal tracking	HoofmApp; Orion Horse Shoes [114,115,116,117]	Early/mostly non-automated

**Table 2:** **Smartphone as a gateway for wearable and external sensor systems in equine sports medicine:** In this configuration, the smartphone functions as an interface for data acquisition, visualization, and integration of external devices. Applications span locomotor, cardiovascular, respiratory, thermoregulatory, and regulatory monitoring domains, with varying levels of technological maturity and clinical adoption. Maturity levels reflect a qualitative assessment based on the availability of peer-reviewed validation studies, degree of clinical or field adoption, and technological development/commercial availability. Technologies classified as High are supported by multiple validation studies and are routinely used in clinical or sporting contexts. Moderate technologies have undergone preliminary validation and show increasing practical adoption but require additional evidence or wider implementation. Emerging technologies are characterised by limited validation data, early-stage implementation, or restricted use in research settings. Early technologies remain largely exploratory and require substantial further validation before routine clinical application. Representative examples were selected to illustrate the principal technological approaches currently described in the peer-reviewed literature. Specific commercial products are reported when they have been evaluated in published studies or are widely cited within the scientific literature. In application areas where individual products have received limited scientific evaluation, broader technology categories are reported instead.

Domain	Smartphone-Integrated Modality	Main Application	Examples (Available Systems)	Maturity Level
Locomotor system	IMU-based wearable sensors interfaced with smartphone applications	Objective gait analysis, movement asymmetry detection, lameness support, longitudinal locomotor monitoring	EquiGait^®^; Equi-Pro^®^; Move Pro^®^ [41,119,120,121,122,123]	High
	Limb-mounted wearable sensors/smart boots interfaced with mobile apps	Distal limb kinematics, localized biomechanical assessment, early detection of limb-specific abnormalities	Tendiboots™ [123]	Emerging
Cardiovascular system	ECG-based wearable systems connected to smartphone apps	Heart rate monitoring, HRV analysis, workload and fitness assessment	Polar Equine^®^; Hylofit^®^ [125,126,130]	High
	Multimodal wearable systems (ECG + GPS + accelerometry) with mobile integration	Integrated physiological and performance monitoring (HR, speed, workload)	Equimetre^®^ [127]	High
	Smart textile-based systems interfaced with smartphones	Continuous ECG acquisition, HRV, autonomic nervous system monitoring	Skiin Equine^®^; Smartex^®^ [128,129]	Emerging
	Portable ECG devices connected to smartphones	Cardiac rhythm assessment, arrhythmia detection (mainly at rest)	AliveCor^®^; eKuore^®^ [131,132]	Moderate
	Smartphone-integrated digital stethoscope systems	Combined auscultation and ECG-based cardiac assessment	Eko DUO [133]	Emerging
Respiratory system	Smartphone-connected borescopes	Upper airway visualization, screening of respiratory disorders	Smartphone-compatible borescope systems [134]	Emerging
Thermoregulatory system	Smartphone-connected infrared thermal imaging devices	Skin temperature assessment, detection of localized thermal changes, heat stress monitoring	Smartphone-compatible thermal cameras [136,137,138,139]	Moderate
Regulatory and monitoring	Digital identity and compliance platforms	Horse identification, passport verification, eligibility and traceability	FEI HorseApp [140,141]	High
	Smartphone-based vaccination monitoring systems	Vaccination compliance tracking, biosecurity monitoring	FEI digital vaccination systems [142,143]	High

**Table 3:** **Comparative overview of the current validation status and measurement reliability of smartphone-based technologies across major physiological domains in equine sports medicine**. The table summarizes the relative strength of available validation evidence and the principal factors influencing measurement accuracy for each application area. Owing to differences in sensing modalities, outcome variables, and validation methodologies, the comparison is intended to provide a qualitative overview rather than a direct quantitative ranking of technological performance.

Physiological Domain	Validation Evidence	Principal Limitations
Locomotor	Strong	Acquisition conditions, algorithm dependence, biological variability
Cardiovascular	Strong	Motion artefacts, sensor placement, signal quality
Respiratory	Limited	Environmental noise, movement artefacts, limited validation studies
Behavioural/Pain assessment	Moderate	Dependence on image quality, dataset bias, algorithm training
Ophthalmic	Limited	Small validation datasets, limited clinical studies
Thermography	Moderate	Environmental influences, poor correlation with core temperature

## Data Availability

No new data were created or analyzed in this study.

## Abbreviations

The following abbreviations are used in this manuscript:

AI	Artificial Intelligence
ANT+	Advanced and Adaptive Network Technology Plus
BLE	Bluetooth Low Energy
CMOS	Complementary Metal–Oxide–Semiconductor
ECG	Electrocardiography
FEI	Fédération Équestre Internationale
GDPR	General Data Protection Regulation
GNSS	Global Navigation Satellite System
GPS	Global Positioning System
HGS	Horse Grimace Scale
HR	Heart Rate
HRV	Heart Rate Variability
IBI	Inter-Beat Interval
IMU	Inertial Measurement Unit
IoT	Internet of Things
mHealth	Mobile Health
MEMS	Microelectromechanical Systems
PPG	Photoplethysmography
